# Rheological Analysis of the Structuralisation Kinetics of Starch Gels

**DOI:** 10.3390/molecules26133826

**Published:** 2021-06-23

**Authors:** Ryszard Rezler

**Affiliations:** Department of Physics and Biophysics, Faculty of Food Science and Nutrition, Poznań University of Life Sciences, Wojska Polskiego 38/42, 60-637 Poznań, Poland; ryszard.rezler@up.poznan.pl

**Keywords:** bispiral chains, functionality, gel, retrogradation, starch

## Abstract

Using the method of dynamic–mechanical analysis, the structuralisation kinetics of condensed starch solutions, cooled down to the temperature of 20 °C, was investigated. A close correlation of spatial crosslinking with local processes of macromolecule associations was discovered. It was found that depending on the concentration intervals of starch solutions, equilibrium nodes of the spatial network assume the form of either single or double hexagonal structures made up of bispiral chain associates. The increase of gel crosslinking, together with the passage of time, is the result of increasing the node functionality of the spatial network.

## 1. Introduction

Starch is one of the most common natural polymers. That is why the main objective of basic research on starch as well as of technologically oriented papers is the elaboration of methods of optimal utilisation of this raw material and establishment of foundations allowing prognostication and control of the course of technological processes aimed at obtaining products of desirable, planned-in-advanced properties. One of the major objectives of the basic research conducted so far on starch has been to recognise its structure in its native state. In this condition, starch is found in the form of spherulitic granules distinguishing themselves by radial anisotropy. The high degree of orientation in starch spherulites of its polymolecular constituents, linear amylose and branched amylopectin, is the source of the optical double refraction manifesting itself when observing starch grains in polarised light [1,2]. Since the time when Katza and Van Itallie [3] carried out their roentgenographic investigations, native starch is attributed with possessing two basic types of crystalline structures: A and B as well as a rarely observed polymorphic C form. A double-helix structure proposed by Kainuma and Frencha [4] is assumed as the most probable model of starch crystalline forms. The chain conformations in A and B structures are identical and dextrorotatory, and the main difference in the structure of both polymorphic forms is that in the A-type structure, the centre of the hexagonal packing is occupied by a seventh double helix, while in the B-type structure, it is empty. The A form crystalline structure is observed in cereal starch (e.g., wheat, rice, and maize); whereas the B form is characteristic for fruit, tuber, and stem starch (e.g., potato and banana). Both A and B structures have parallel, dextrorotatory bihelical conformations of chains packed in cluster forms [5,6].

Crystalline forms of the second starch constituent—amylopectin—can be attributed to the identical bihelical organisation of branches to that described above [7] packed in forms of clusters [8,9].

The second branch of the physicochemical direction of research of starch systems, which is currently developing very strongly, is the problem of structure changes of the discussed systems in conditions of the hydrothermal processing frequently accompanying culinary processes. During the heating of water starch suspensions to temperatures of over 50 °C, starch spherulites swell and lose their optical anisotropy, which is identified with the melting of the polymer crystalline phase. Complicated structural changes taking place during the melting of crystalline starch areas are subjected to interpretations from the thermodynamic point of view [10,11,12] based on Flory’s [13] statistical theory.

Starch solutions obtained in normal cooking conditions of suspensions show tendencies of constituent aggregation following their cooling and, at sufficiently high concentration, the capability to develop a permanent spatial structure [14,15].

The aggregation process of starch structural elements taking place in the described conditions, which is frequently accompanied by a liquid phase as a result of synthesis, is traditionally referred to as retrogradation. Due to the fact that retrogradation affects many quality parameters of almost all products of starch processing, investigations of a complex of phenomena accompanying starch retrogradation constitutes the subject of many analyses and monographic studies, in particular, those that aim at elucidating the mechanism of bread staling [9,16,17].

Already, the results of the first roentgenographic investigations [3] have revealed that one of the effects of starch structure changes during the retrogradation process is its partial crystallisation. Further studies carried out using both roentgenographic [18,19], NMR [20,21], as well as calorimetric [22,23] methods confirmed this opinion and created foundations for the quantitative characterisation of the retrogradation process on the basis of the phenomenological theory of crystallisation [24]. However, the performed rheological investigations [25,26,27] indicate that despite a certain correlation with changes in the degree of starch system crystallisation, it is the dispersive structure of amorphous regions that decides the mechanical properties of these systems that have important practical significance. It can be presumed that the retrogradation changes taking place within these regions, as the ones most accessible for substances accompanying starch in food products, will affect not only changes in consistency but also changes in the taste, flavour, and colour of these products due to the evolution of starch potentials to bind substances responsible for the above-mentioned food quality parameters.

## 2. Results and Discussion

In order to characterise the molecular preconditioning of the starch retrogradation process, measurements were performed of the structuring kinetics of starch solutions cooled down to the temperature of 20 °C in an atmosphere of saturated water vapour, using for this purpose the DMA method. This allowed the determination of components of the complex modulus of elasticity of the developing spatial network and assessment of the density of the segments of macromolecules making up the spatial network.

**Figure 1:**
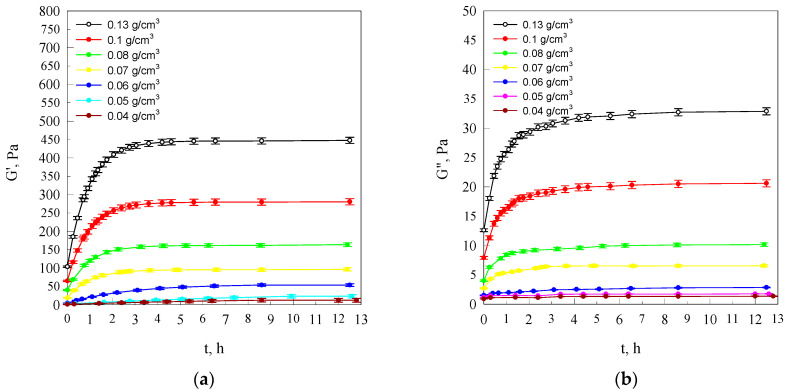
Kinetics of storage modulus (*G*′) (**a**) and of the loss modulus (*G*″) (**b**) in the process of formation of starch gels at different concentrations of starch in systems. The values are expressed as means ± SD (*n* = 3).

The character of the dynamic–mechanical changes of the starch solution properties taking place with the passage of time, counted from the moment of reaching ambient temperature at various concentrations, illustrates the model courses of the dependences *G*′(*t*) and *G*″(*t*) presented in Figure 1.

With the passage of time, both storage modulus (*G*′) and loss modulus (*G*″), at different concentrations, increase, tending towards asymptotic values.

According to Clark and Ross-Murphy [28], the initial increase of modulus *G*′ and modulus *G*″ can be attributed to the development of a three-dimensional network following associations between amylose chains, whereas the slow increase of *G*′ and *G*″ is the result of the formation of additional cross linkages and further aggregation of the polymer chains. Such value changes of components of the complex modulus of elasticity are the result of the advancing process of amylose retrogradation [29].

The growth increment of the storage modulus (*G*′) and loss modulus (*G*″) values increases together with the increase in the starch concentration. This is due to the fact that the retrogradation process is faster at more concentrated systems of starch dispersion than in a dilute dispersion [29,30].

In addition, the attainment of plateau values for both *G*′ and *G*″ was much higher in gels of high content starch (Figure 1). This is the result of the development in the examined systems of a spatial network of varying segment concentration, which is dependent on starch concentration. On the basis of the equilibrium *G*′ values not exceeding several hundred Pascals, it can be said that the totally hydrated starch chain fragments making up segments of the spatial networks of the examined starch hydrogels are in a highly elastic state. Initial values of storage modulus *G*′ different from zero indicate that during the cooling process, a spatial network is already developing in the examined systems with the segment concentration *n*(*c*) dependent on starch concentration. The approximate relationship between the storage modulus of highly elastic polymer networks and the segment concentration in these networks is determined by the following dependence [31]:

(1) $$n≅\frac{G^{\prime }}{RT}.$$

The observed increase of the hydrogel storage modulus together with the passage of crosslinking time at different concentrations indicates that the concentration of effective segments in this network increases as asymptotic values of *n_∞_*(*c*) are approached. These changes can be described by the Avrami-type [24] Equation (2):

(2) $$n(t)=n_{o}+(n_{\propto }-n_{o})(1-\exp[-{\left(kt\right)}^{m}])$$

where *n_o_* and *n_∞_* designate initial and final network segment concentrations, *k* is the Avrami crystallisation rate constant, and *m* is the Avrami exponent of time.

The parameters *m* and *k* of the Avrami equation characterised the mechanism of nucleation, geometry of the crystallisation, and growth of crystallites. Constant *k* characterised a crystal growth rate dependent on the temperature and molecular weight [25,32,33].

The Avrami exponent (*m*) according to the theory is an integer value ranging from 1 to 4 (depends on the type of nucleation, geometric forms, and crystal growth mechanism) [32].

In crosslinked systems, the bond density and durability exert a decisive influence on their mechanical and rheological properties; hence, the change kinetics of the *G*′ and *G*″ components in the course of the observed crosslinking can be described by the equation of the same type:

(3) $$G(t)=G_{o}+(G_{\propto }-G_{o})(1-\exp[-{\left(kt\right)}^{m}])$$

where *G_o_* and *G_∞_* designate, respectively, initial and final values of storage modulus.

**Figure 2:**
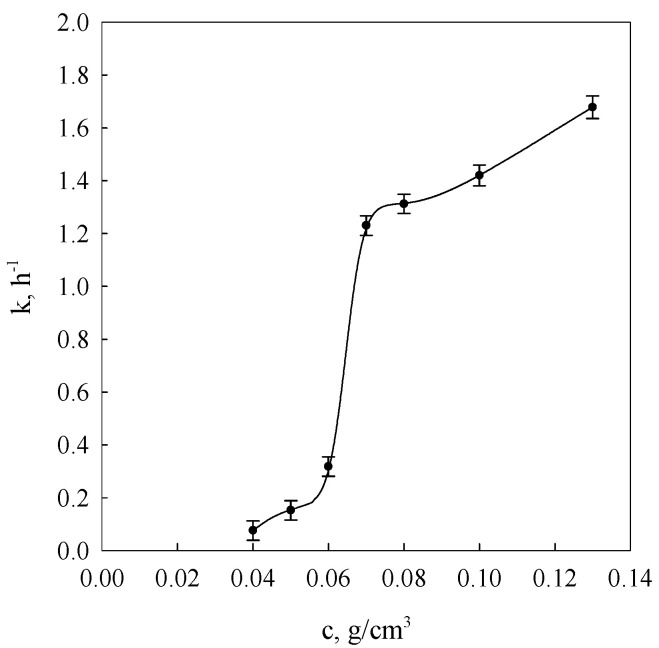
Concentrational relationships of crosslinking kinetics constants (*k*) of starch gels at different concentrations starch in systems. The values are expressed as means ± SD (*n* = 3).

Figure 2 presents concentrational relationships of kinetics constants arrived at by fitting data obtained using the DMA method into Equation (3).

It was found that the Avrami exponent m assumes the value of 1 throughout the examined interval of starch concentrations.

This appears to suggest that the node formation processes of the spatial network of the examined gels attain only the nucleation phase of crystalline forms.

The character of the constant *k* changes (Figure 2) indicates a diversification of molecular processes determining the range and rate of development of the gel spatial network nodes. As evident from earlier considerations, in such systems, links between different fragments of macromolecules neighbouring with one another in solutions act as sources initiating the establishment of spatial network nodes (spiralisation and association to bihelical forms of associated molecules). In conditions of low polymer concentration in the solution, only the coordination macromolecular spheres, consisting of long amylose chains and long branched chains of amylopectin can overlap, leading to their spiralisation and association to bihelical forms. In the case of higher concentrations from 0.06 g/cm^3^, short amylose chains as well as short branched chains of amylopectin also participate in the development of spatial network nodes [34,35,36].

A very similar course of *k*(*c*) constant relationship was obtained as a result of the relaxation of velocity measurements of identical wheat starch gels, using for this purpose the NMR technique [37,38] in a range of concentrations exceeding 0.06 g/cm^3^. This indicates a close correlation of spatial structuring with local association processes.

In the case of polymer systems of an established spatial network containing no individual polymer molecules, according to the statistical–thermodynamic theory [13], the chemical potential of the diluent is determined by the following dependence:

(4) $$\mu^{\prime }-\mu^{o}=RT[\ln(1-v_{2})+v_{2}+\chi {v_{2}}^{2}+v_{1}(v_{2}^{-\frac{2}{3}}-\frac{2}{f})n]$$

where *μ^ο^* is the chemical potential of a pure diluent, *v*_1_ is a volumetric share of the diluent, *v*_2_ is a volumetric share of the polymer in the system, *χ* is a parameter of the polymer–diluent interaction, *n* is the number of segments of a volume unit of swelled gel, and *f* is the node functionality of the spatial network of the gel.

When the process takes place in the presence of diluent vapours, then the chemical potential of this phase is determined by the following expression:

(5) $$\mu^{\prime }-\mu^{o}=-RT\ln(\frac{p}{p_{o}}).$$

When a gel spatial structure is formed in the atmosphere of the diluent saturated vapour, then its pressure over the solution *p = p_o_.* Therefore, the condition for the stabilisation of the chemical potential equilibrium requires evolution of the constituent values of Equation (4), leading to neutralisation of the left side of this equation:

(6) $$\mu^{\prime }-\mu^{o}=0.$$

This can take place not only following changes in the spatial network segment concentration and their functionality but also as a result of the increased effectiveness of polymer concentration combined with decreasing the volume taken up by this polymer. The phenomenon of the release of pure diluents accompanying the process, known as syneresis, is a frequent manifestation of the starch retrogradation process [39]. Therefore, the content of network segments in a volume unit of gel is determined by the following expression:

(7) $$n=-\frac{\ln(1-v_{2})+v_{2}+\chi {v_{2}}^{2}}{v_{1}(v_{2}^{-\frac{2}{3}}-\frac{2}{f})}.$$

**Figure 3:**
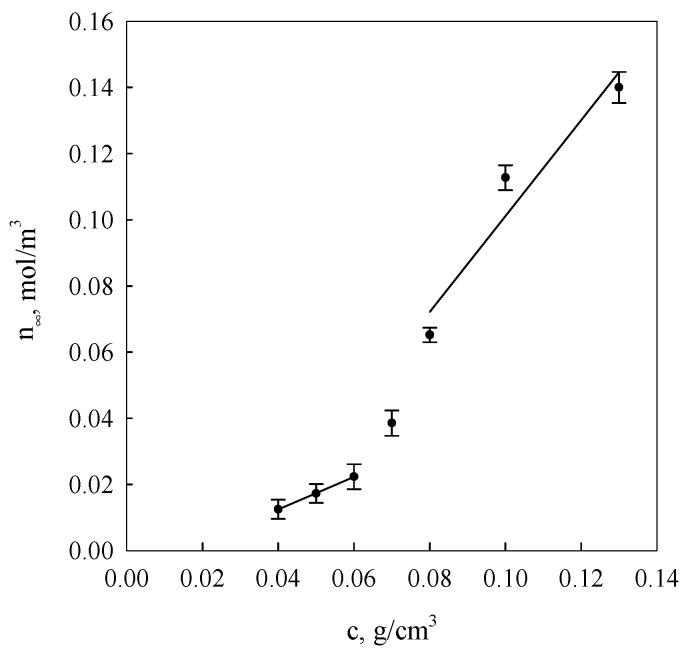
Concentrational relationships of starch gel segment equilibrium concentrations (*n_∞_*) at different concentrations starch in systems. The values are expressed as means ± SD (*n* = 3).

Figure 3 presents courses of segment equilibrium concentrations n_∞_ in the polymer concentration function in the solution calculated on the basis of Equation (2) as well as curves obtained in the result of additional fitting of these courses to Equation (7).

**Figure 4:**
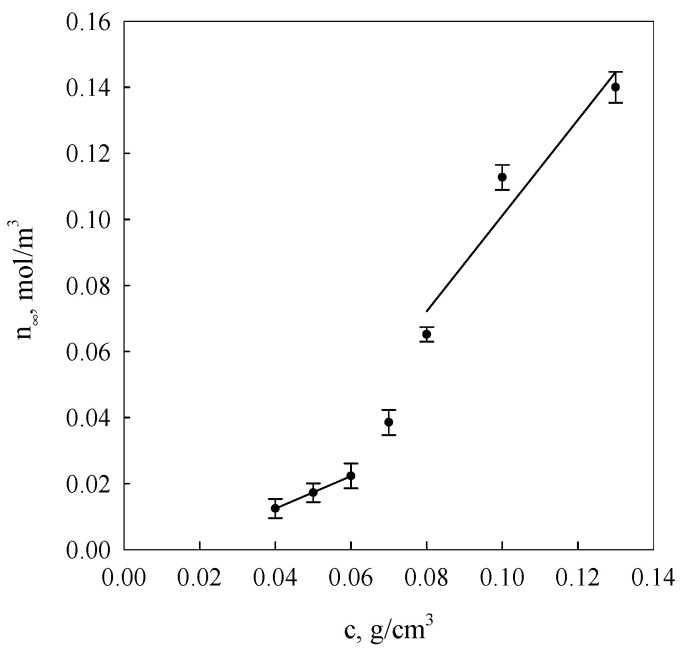
Concentrational relationships of equilibrium node functionality values (*f*) of starch network gels at different concentrations starch in systems. The values are expressed as means ± SD (*n* = 3).

Figure 4 presents the change of network node functionality of the examined gels.

It is evident from the data analysis presented in Figure 3 and Figure 4 that gel dispersion structures formed as a result of starch retrogradation in solutions with concentrations below 0.06 g/cm^3^ and over 0.07 g/cm^3^ show distinctly greater diversity. The initial gel structure at low starch concentrations is determined by nodes of spatial networks that occur, primarily, in the form of single hexagonal structures made up of bispiral chains of associated molecules, i.e., nodes of functionalities of the order of 30. In the case of aggregate organisation in characters corresponding to the polymorphic type A form, their functionality should assume the value *f* = 28. Then, it follows that the structures of the re-organised regions in retrograded starch differ from those in native starch. B-type crystals are found in retrograde cereal starch [29,40] that showed an A-type structure before gelatinisation. Once the concentration of 0.06 g/cm^3^ is exceeded, a discrete change of network node functionality of the examined gels takes place, indicating the possibility of the development of hexagonal binary structures. This was also corroborated in the research results of earlier publications [25,38] in which it was demonstrated that for the processes of starch gel spatial structuring, changes in the association enthalpy and entropy assumed values were more than twice as high as those determined for spiralisation processes of single starch chains. These data indicated a cooperativeness effect of a bispiral association involving six-link coils affecting fragments of polymer chains.

In conditions of unstable gel spatial structure when, as a result of crosslinking, network segment concentration and the functionality of its nodes can change, the analysis of the state of the starch–water system cannot be carried out according to the method proposed above. It is only possible to perform an approximate analysis on the basis of the interdependence between network segments n, node concentration w, and their functionality *f:*

(8) n=0.5wf

and taking into consideration assumptions of the phenomenological theory of polymer crystallisation kinetics [31], according to which the polymer segment adsorption kinetics of node nuclei of a spatial network can lead to increased functionality of these nodes in accordance with the following equation:

(9) $$f=f_{o}+(f_{\propto }-f_{o})(1-\exp[-{\left(kt\right)}^{m}])$$

where *f_o_* and *f_∞_* designate, respectively, initial and equilibrium functionalities of nodes; k is the kinetics constant; and m is the Avrami constant.

Then, Equation (2) can be presented in the following form:

(10) $$n(t)=0.5w\{f_{o}+(f_{\propto }-f_{o})(1-\exp[-{\left(kt\right)}^{m}])\}.$$

**Figure 5:**
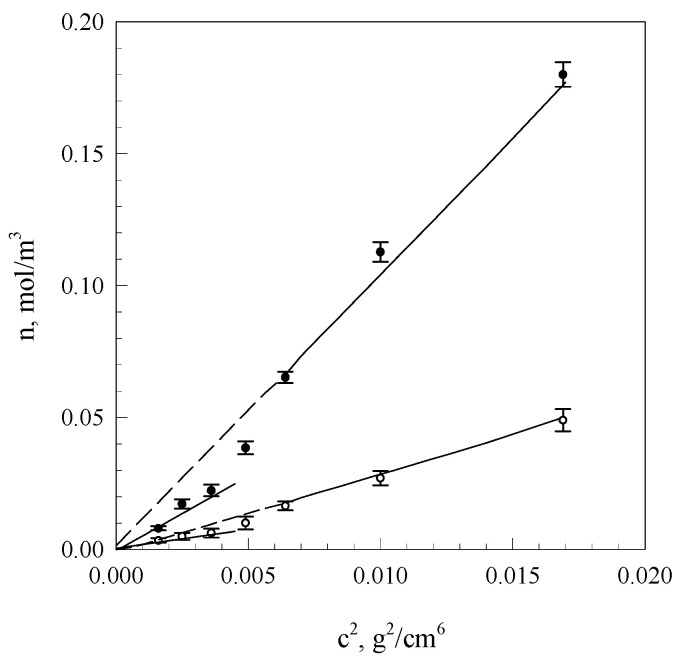
Interdependences of initial (◦—*n_o_*) and equilibrium (•—*n_∞_*) gel network segment concentrations in the function of squared starch concentration in the system. The values are expressed as means ± SD (*n* = 3).

Using the method of computer fitting, it can be demonstrated that assuming constant node functionality, the increase of polymer concentration in a crosslinked gel should lead to the increase of network node concentration in accordance with the dependence: *n*~*c*^2^ (*c*-polymer concentration). On the other hand, when the node concentration is assumed as a constant, the increase of polymer concentration will cause an increase of the average functionality proportional to *c.* The diagrams shown in Figure 5 indicate that equilibrium (*n_∞_*) and initial (*n_o_*) network segment concentrations are subject to linearisation in function *c*^2^, adopting, however, slopes that depend on the system condition and starch solution concentration interval below and above *c* *≈* 0.06 g/cm^3^.

These data suggest that with gel aging, fragments of starch macromolecules, gradually incorporated into the network, undergo adsorption on these nodes, increasing their functionality. It is evident from the obtained values of node functionality of the equilibrium network and from the ratio of directional coefficients of anamorphoses presented in Figure 5 that the initial mean functionality of network nodes assumes values dependent on the interval of starch network concentrations below and above *c* ≈ 0.06 g/cm^3^ i.e., respectively, 3.7 and 26.5. This means that within the range of low starch concentrations, the initial gel structure is determined by tetrafunctional nodes that are bispiral chain associates, whereas within the range of concentrations above 0.06 g/cm^3^, hexagonal associates of bispiral structures are involved. The discrete change of node functionality observed at starch concentration of 0.07 g/cm^3^ is probably connected with the occurrence of areas of spatial arrangement that are characteristic for solutions of liquid crystal polymers [13]. The occurrence of different conformation states of starch chains finds an increasingly wider application in the interpretation of experimental results [41,42].

It is evident from earlier considerations that the formation of a spatial network in starch gels is the result of random linkages between different fragments of macromolecules neighbouring with one another in the solution, leading to spiralisation and their association to forms of bihelical associates. Therefore, individual nodes of the gel network are characterised by varying functionalities assuming values from about 4 to several dozen.

This is associated with the energy differentiation of *ΔU_i_* segment linkages in individual nodes and, consequently, leads to the distribution of mechanical relaxation times resulting from the following dependence:

*τ_ι_ = τ_ο_ exp[ΔU_i_/(RT)]*. (11)

The mechanical–rheological properties of polymers are conditioned by the function course of the relaxation times distribution [43]. Therefore, it is appropriate to adopt for gels a two-dimensional distribution of relaxation times that characterises the highly elastic state of the elastic molecule network [31].

Hence, the standard function of the distribution of relaxation times can be approximated by the rectangular distribution for which:

(12) $$H(ln\tau )/\delta G\;=\;\frac{1}{\ln(\frac{\tau_{G}}{\tau_{D}})}$$

in the interval between the bottom *τ_D_* and top *τ_G_* spectral limits, while outside this interval, *H(lnτ)/δG* = 0.

At sufficiently broad distributions and frequencies *ω* ≅ 1/*τ_average_*, it can be assumed [43] that.

(13) $$\frac{G^{\prime \prime }}{G^{\prime }}=\frac{\pi /2}{\ln(\frac{\tau_{G}}{\tau_{D}})}.$$

Using the empirical *G*′ and *G*″ values, it is possible to determine the energetic state of the gel network as well as changes of this state during the structuring process. Taking Equation (11) into consideration, Equation (13) assumes the following form:

(14) $$\frac{G^{\prime \prime }}{G^{\prime }}=\frac{\pi RT}{2(\Delta U_{G}-\Delta U_{D})}$$

where (Δ*U_G_* − Δ*U_D_*) = *δU* is the difference of the activation energy of the relaxation processes accompanying the deformation of the gel network segments connected with the strongest and weakest nodes of this network.

It is evident from the data shown in the drawings in Figure 1 that this difference increases together with the structurisation process assuming values in the interval of (20–115 kJ/mol) for *c ≤* 0.06 g/cm^3^ concentrations and values from approximately 30 to 60 kJ/mol for starch concentrations exceeding 0.06 g/cm^3^.

The author applied successfully the same procedure in the study [38] in order to determine the linkage energy distribution of the network segments and to ascertain thermodynamic parameters (changes of the ΔH enthalpy and ΔS entropy) taking place during the formation process of a spatial network of the examined gels in the course of storage at different temperatures.

## 3. Materials and Methods

### 3.1. Materials and Sample Preparation

Investigations were carried out on starch hydrogels obtained from starch solutions of *Triticum durum* wheat (International Grain Products, Canada). The chemical composition of wheat starch was as follows: moisture, 12.2%; protein, 0.44%; lipid, 0.39%; ash, 0.21%; and 90% amylopectin. The starch used in the studies was dried to a constant mass at the temperature of 100 °C prior to the preparation of solutions. Starch dispersion samples weighing 100 g were subjected to thermal treatment at a constant temperature of 90 °C, in an LWC2M water bath (DANLAB Company, Switzerland) for a period of 1 hour and stirred continuously with the assistance of an IKA EUROSTAR agitator power control-visc 6000 of IKA WERKE GmbH (Staufen Germany). Solutions of 0.04 to 0.13 g/cm^3^ starch concentration obtained in this way were used to fill the measuring system of the rheological analyser and cooled to the required temperature with the velocity of 4 °C/min.

### 3.2. Mechanical–Rheological Properties

A dynamic mechanical thermal analyser (DMWT) (COBRABiD-Poznań, Poland) was used for measurements. A parallel plate with a 50 mm diameter and 1.0 mm gap measuring system was used. The sample perimeter was covered with a thin layer of high-temperature-resistant silicone grease (GE Electronics, Rockford, IL) to prevent dehydration of the sample edge and moisture evaporation from the sample. The following components of the complex modulus of elasticity were calculated: storage modulus (*G*’) and the loss modulus (*G*″). The storage modulus is associated with the part of potential deformation energy that is maintained in the course of periodical deformations. Loss modulus (*G*″) is associated with the part of energy that undergoes dissipation in the form of heat. The analyses were performed considering the linear viscoelastic region in each sample. The frequency of the vibrations of the systems amounted to 1.2 Hz. Measurements of the starch structuralisation kinetics were carried out at a set temperature of 20 °C (with up to 0.2 °C accuracy).

### 3.3. Statistical Analysis

The mean and standard deviations were calculated from the rheological measurements on three newly prepared samples in accordance with the procedure described earlier. The data presented on Figures are the result of averaging of the results obtained in individual replications. Mean values as well as standard deviations (*SD*) were calculated with the assistance of the Statistica 10.0 PL software. The values of changes of *m*, *k*, *f*, and *n* parameters were obtained by the method of computer fitting (least squares method, TableCurve 2D).

## 4. Conclusions

In order to characterise rheological attributes of the examined starch–water systems, the analysis of kinetic changes of basic parameters determining their mechanical–rheological properties was carried out. The research results of starch gel systems obtained using the DMA method indicate that with the passage of time counted from the moment they reach ambient temperature, a spatial network with segment density dependent on starch concentration develops in the examined systems already during the process of cooling down. It is worth mentioning that the kinetics of the run of crosslinking processes conforms to the description of the Avrami-type equation. The kinetic parameters of crosslinking that determine the extent and rate of spatial network node development in gel systems, similar to the initial and final densities of network segments, are preconditioned by the concentration of starch in the system.

It is evident from the dependence between segment concentration in the gel network and node concentration as well as the functionality of these nodes that at a given starch concentration in the system and at constant temperature, the increase of gel stiffness in time function occurs as a result of node expansion involving the binding of new macromolecule segments. This leads to the increase of node mean functionality stabilising the spatial network.

## Data Availability

The data presented in this study are available on request from the author.
